# 

**Published:** 1879-09-20

**Authors:** 


VOL. IX. SEPTEMBER 20, 1879. NO. 9

### THE SOUTHERN MEDICAL RECORD:

A MONTHLY JOURNAL OF PRACTICAL MEDICINE.

Terms: $2 00 per Annum, in Advance; 4 Copies $6.00; Single Copies 20 Cents.

*"QUICQUID PRÆCIPIES ESTO BREVIS."*

Address R. C. WORD, M.D., Managing Editor, Atlanta, Ga.

